# Ocean Surface Drifting Buoy System Based on UAV-Enabled Wireless Powered Relay Network

**DOI:** 10.3390/s20092598

**Published:** 2020-05-02

**Authors:** Haole Chen, Feng Yin, Wei Huang, Mingliu Liu, Deshi Li

**Affiliations:** 1 Electronic Information School, Wuhan University, Wuhan 430027, China; chenhaole@whu.edu.cn (H.C.); whuwsnhw@whu.edu.cn (W.H.); liumingliu@whu.edu.cn (M.L.); 2 School of Science and Engineering, The Chinese University of Hong Kong (Shenzhen), Shenzhen 518172, China; yinfeng@cuhk.edu.cn; 3 Shenzhen Research Institute of Big Data, Shenzhen 518172, China

**Keywords:** buoy communication, unmanned aerial vehicle (UAV), wireless powered communication networks, resource allocation, maximize minimum throughput

## Abstract

We design an ocean surface drifting buoy system based on an unmanned aerial vehicle (UAV)-enabled wireless powered relay network in which the UAV acts as mobile hybrid access point that broadcasts energy to all buoys in the downlink and forwards information from the buoys to a ship signal tower (ST) in the uplink. In order to maximize the resource allocation efficiency of the system, due to the different initial energy reserve of the buoys, a novel communication mode selection strategy is proposed. In the direct transmission mode (DT mode), an energy-sufficient buoy transmits information directly to the ST, and in the relay transmission mode (RT mode), an energy-insufficient buoy relays information to the ST through the UAV. By applying the block coordinate descent and successive convex optimization, a joint UAV trajectory and resource allocation algorithm is proposed to maximize the minimum throughput of the buoys to work in the RT mode. Simulation results show that the proposed algorithm can significantly improve the minimum throughput of the ocean surface drifting buoys.

## 1. Introduction

As one of the most important ways for humans to explore the ocean, the development of ocean data buoys (ODBs) can be traced back to the 1940s [1]. The surface drifting buoy is a kind of small ODB and mainly uses various types of sensors to obtain the relevant data in the surrounding marine environment for ocean investigations, environmental monitoring, and scientific experiments [2]. To obtain the data collected by the buoys, there are currently two main modes of buoy communication: satellite communication (such as maritime satellites, the Beidou satellite navigation system, and the Argo satellite communication system) and autonomous underwater vehicle (AUV) communication. Although satellite communications can provide a good solution for buoy communication in deep-sea exploration, there are a number of shortcomings (e.g., low transmission rate, low reliability, and high communication cost), and the use of satellite communications offshore, in inland lakes, and other areas with frequent ship activities may result in a waste of resources. In addition, due to the slow sailing speed of the AUV, the delay of data transmission is high, which means AUV–buoy communication cannot meet real-time data requirements, and the maintenance and recovery of the AUV will introduce a series of new difficulties.

Compared to traditional AUV communications or satellite communications, unmanned aerial vehicle (UAV)-enabled communication networks can reduce networking costs and allow rapid deployment [3]. In addition, the line-of-sight (LOS) communication links between a UAV and the buoys usually provide exceptional channel quality and, consequently, more stable and reliable communication services [4,5]. Furthermore, due to the rapid development of the UAV industry and technology, UAVs are now available in all shapes and sizes, ranging from long-range, high-durability models to small portable UAVs with short surveillance range [6], which means UAV communication has the potential to become an efficient and energy-saving solution for a buoy communication system. The authors of [7,8,9,10] explored the application of UAV in the ocean; the authors of [7] proposed a UAV and AUV coordination mechanism for ocean exploration; the authors of [8] considered employing UAV as an element of an ocean observing system; the authors of [9] designed UAV surveillance frameworks for extensive ocean; the authors of [10] designed a small UAV that can fly continuously over the ocean.

UAV-enabled communication networks mainly use UAVs as mobile micro-base stations/relays/access points to collect/transmit information from/to wireless terminals. The works in [11,12,13,14,15] consider the application of UAVs in different communication scenarios and provide an optimization scheme for the UAV trajectory and resource allocation. An efficient successive convex-approximation (SCA) technique was proposed by Zeng et al. in [11] to optimize the UAV trajectory and communication resource allocation. Based on the SCA technique, the authors of [12] considered a UAV-enabled date collecting system with multiple users, and maximized the average rate by jointly optimizing the flight trajectory of the UAV and the information transmission power of the user. The authors of [13] studied a UAV-enabled mobile relay network and the UAV trajectory and time allocation were jointly optimized to improve the energy efficiency. In [14,15], an efficient iterative algorithm was proposed to optimize the UAV trajectory and transmission power in turn for a multi-UAV communication network. Although the UAV needs to forward the collected information to the background operator through the base station, the communication link between the UAV and the base station is not considered in the above references. In addition, as the communication duration and information transmission rate of devices in the network are severely limited by the current energy condition, the energy status of users and UAVs in energy shortage buoy communication scenarios should be further analyzed.

In general, buoys are primarily powered by internal batteries, and increasing the number or volume of batteries is the simplest way to prolong the working life of the buoys. However, increasing the size and weight of the battery not only increases the cost of deploying the buoy, but also causes a series of problems with battery replacement and maintenance. Therefore, the question of renewable energy for ocean buoys has been a key issue since modern research on the topic emerged in the early 1970s [16]. Solar energy and wave energy are the two main ways for buoys to obtain renewable energy [17].

However, due to the instability of renewable energy collection and the high construction cost of collection devices, this approach is not suitable for large-scale use in most sea areas. Furthermore, although this renewable energy can supplement the energy of buoys during long-term intermittent work, the energy requirements of buoys in emergency situations or real-time communication scenarios cannot be guaranteed. In contrast, the interference-free LOS channel in the buoy–UAV communication network provides an ideal environment for radio frequency (RF) energy signal transmission [5], which means that a UAV–buoy communication network based on wireless energy transfer (WET) technology can be considered as a promising solution.

Wireless communication networks based on WET technology have been widely considered in terrestrial scenarios [18,19,20], and the wireless powered communication network (WPCN) is one of the representative technologies [21]. A typical WPCN employs a hybrid access point (H-AP) that acts as a power station to broadcast RF energy signals to the ground terminals during the downlink and as an information receiver station in the uplink. However, the so-called “double near-far” phenomenon has long existed in WPCN: the user close to an H-AP can harvest more energy in the downlink and experiences a better channel condition in the uplink than a user far from the H-AP.

Several WPCN models have been proposed to overcome the above near-far fairness problem. The authors of [22] consider an additional constraint that guarantees the throughput required by the user through optimal allocation of time resources regardless of the channel condition between users and the H-AP. Our previous work [23] proposed a relay strategy with an incentive mechanism, which means that users closer to an H-AP can sell excess energy to help other users forward information. The authors of [24] aim to maximize a user’s throughput by optimizing the number and locations of H-APs. However, limited by the fixed position of the H-AP and user equipment, these existing studies are based on the premise of a constant uplink/downlink channel power gain, which makes it difficult to fundamentally solve the near-far fairness problem.

Several previous works [25,26,27,28,29] have attempted to overcome the above problems by using UAV as a mobile H-AP and constructing UAV-Based WPCN (U-WPCN). The authors of [25] use the UAV as relay node to fly around the fixed ground H-AP with a given radius. In this scenario, the UAV can provide periodic information relay services for energy shortages users; however, because the trajectory and flight speed of a UAV cannot be adjusted according to the user’s specific location, the communication quality of edge users cannot be guaranteed in [25]. The authors of [26,27] studied a hover-and-fly trajectory to optimize the total throughput of users. However, the problem of system throughput maximization includes not only the UAV trajectory but also some other variables such as the UAV flight time, the user’s initial energy and the resource allocation (e.g., energy broadcasting power and information transfer power). Our previous work [28] proposed a user grouping and UAV hovering strategy to improve the system’s performance by deploying multiple hovering UAVs. As the communication coverage of a single UAV in a hover state is limited, our previous work [29] further considers optimizing the flight path of a single relay UAV to cover a larger area to maximize the system throughput while reducing the delay. However, the fairness problem of maximizing the minimum throughput and the problem of joint trajectory and resource allocation including UAV energy broadcast power optimization has not been considered in our previous works. In addition, the optimization sequence and convergence speed in multiobjective optimization problems must be further analyzed. Finally, to the best of our knowledge, the current research on UAV communication systems has not delved into the wireless terminal’s communication mode selection issues, which means that compared to resource-constrained users, those with sufficient initial energy may obtain the same services from a UAV, resulting in a waste of resources.

This paper aims to address the difficulties regarding the energy shortage and information transmission in the offshore surface drifting buoy system. Notably, different from the multinode system with limited energy on the ground [29], the UAV-based information collection and energy broadcasting platform is suitable for a floating buoy communication system. The reasons can be summarized as follows. First, the lower electromagnetic interference and reliable LOS channel enable a UAV to transmit energy to the buoy’s surface communication module more efficiently, which means that a low-complexity linear energy harvesting model can be used [30]. Second, the communication area of the buoys can reach several thousand square meters, and the buoys are scattered and far apart. In this case, the mode selection strategy proposed in this paper can enable the UAV to selectively provide information and energy services for energy-deficient buoys to better utilize the cruise advantages of the drone and improve flight efficiency. Finally, because the sea level has no undulations, the UAV can safely fly at a fixed height. At this time, the UAV’s flight trajectory can be projected onto the two-dimensional plane for optimization, and a reliable flight path can be obtained with less computational effort.

**Figure 1:**
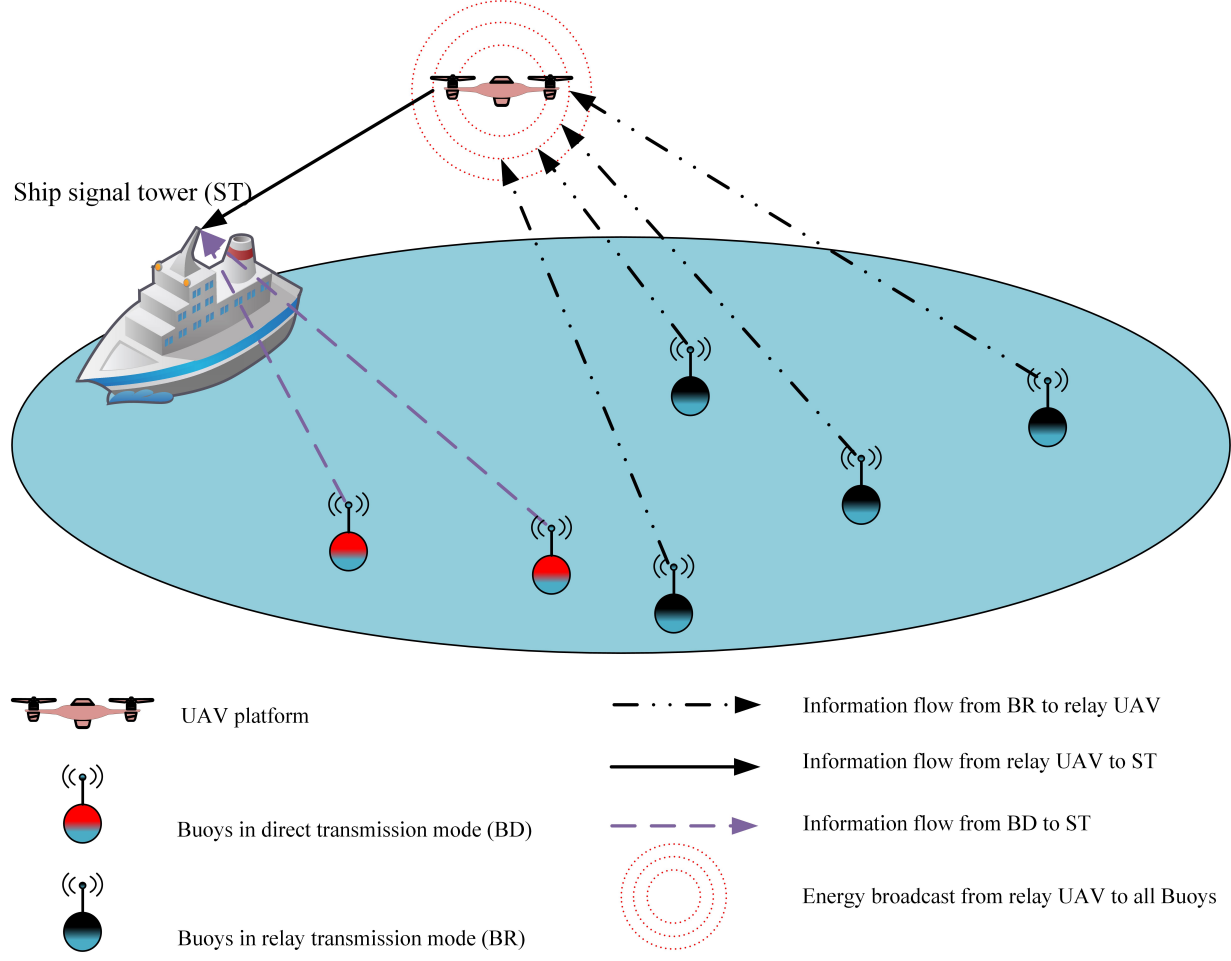
Unmanned aerial vehicle (UAV)-enabled wireless powered relay network (U-WPRN) system model with relay mechanism.

Motivated by the U-WPCN as well as the offshore surface drifting buoy system, this paper pursues a unified study of both of them as shown in Figure 1. We proposed a UAV-enabled wireless powered relay network (U-WPRN) in which the UAV platform is equipped with H-AP, broadcasts energy to the buoy node in the downlink, and forwards the information collected from the buoy node to the signal tower of the working ship in the uplink. To improve the utility of the UAV platform, a buoy communication mode selection mechanism is proposed for determining which buoy requires the help of the UAV to forward information to a ship signal tower (ST). Block coordinate descent (BCD) [31] and SCA technology are used as an algorithm framework [32] to sequentially optimize the UAV trajectory, forwarding power allocation, broadcasting power allocation, time slot allocation, and transfer power allocation of the buoy to obtain a suboptimal solution with low complexity. The main contributions of this paper are summarized as follows.

- *We proposed a U-WPRN for an ocean buoy system.* Unlike the UAV hovering scenarios considered in [26,27,28], this paper first proposed a U-WPRN using a single UAV cruise in the offshore buoy communication scenario. Besides, different from the work in [25], we use the UAV as a mobile relay and let it broadcast energy to buoys to guarantee the communication quality even when the buoy lacks energy. Additionally, different from the work in [29], we considered a decode-and-forward (DF) protocol that can forward information in real-time and ensure the effectiveness of information transmission.
- *We proposed an information transmission mode selection strategy for buoys.* According to the buoy’s initial energy reserve and channel condition, this paper introduces a buoy’s transmission data rate threshold to determine whether the buoy requires UAV relay services during the wireless information transmission (WIT) phase, thus maximizing the energy efficiency of the system. To the best of our knowledge, in the UAV communication network, the communication mode selection strategy based on the user’s current energy and channel conditions is discussed for the first time in this paper.
- *We proposed an optimal resource allocation algorithm for buoys working in the relay mode.* The current research in [11,12,13,14,15,28,29] considered optimizing the UAV trajectory, hovering point, or transmission power for a UAV-ground node communication scenario. In contrast, this paper considers the joint optimization in U-WPRN including subslot allocation, UAV transmission power, buoy transmission power, UAV trajectory, and UAV broadcast power to maximize the minimum throughput of the buoys working in RT mode. Furthermore, by applying the BCD and SCA technique, a joint optimization algorithm with five different suboptimal problems is proposed, and the convergence speed of the algorithm under different optimization sequences is further analyzed to maximize the system performance and improve the UAV’s utility, these are not considered in the above references.

The remainder of this paper is organized as follows. The U-WPRN system model is presented in Section 2. The buoy’s communication mode selection mechanism and problem formulation are given in Section 3. Based on the successive convex optimization algorithm, the optimal UAV trajectory and relay resource allocation are described in Section 4. The numerical results are presented in Section 5, and finally the paper is concluded in Section 6.

## 2. System Model

**Figure 2:**
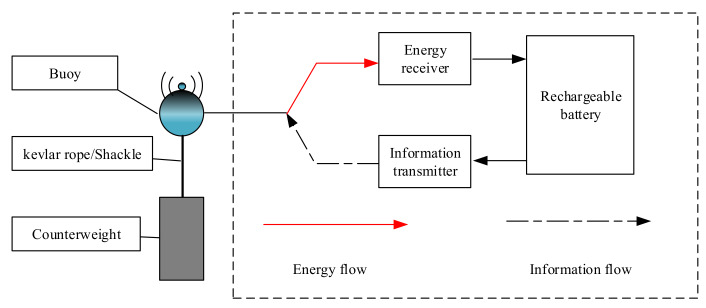
Components of the buoys in our proposed system.

As shown in Figure 1, a U-WPRN system with one ST, one UAV-based H-AP, and several buoys is considered in this paper; the buoy set is denoted as K, $\left|K\right|\geq 1$, and all buoys are equipped with two antennas, one of which is dedicated to energy harvesting and the other one for information transmission; moreover, they do not interfere with each other. To resist the impact of ocean waves and reduce the risk of rollover, this paper considers a spherical buoy with counterweight and the antenna kept above the sea surface. The buoy’s structure is given in Figure 2, and the definitions of all symbols used in this article are shown in Table 1.

In this paper, we consider a UAV equipped with a GPS module for obtaining its own position with high accuracy in a marine LOS environment. In addition, by using cooperative localization techniques [33,34] among the UAVs, the positioning accuracy can be further improved.

Ignoring the height of the buoys, the horizontal coordinate of the *k*-th k∈K buoy is $G_{k}=\left(x_{k},y_{k}\right)$. The height of the ST is assumed to be the same as the fixed flying altitude *H* of the UAV, and the ST is located at the origin of the horizontal coordinate system $O=\left(0,0\right)$. Without loss of generality, the horizontal coordinate of the UAV at time instant *t* is denoted by $U\left(t\right)=\left(x(t),y(t)\right)$, 0<t<T. In addition, in this paper, buoys and ST are considered to be stationary during the UAV flying period *T*. To discretize the trajectory of the drone, the flying period *T* is discretized into *N* equally spaced time slots, and the length of each time slot, $\Delta =\frac{T}{N}$, is chosen to be sufficiently small such that a UAV’s location is approximately unchanged between two consequent time slots even when flying at the maximum speed $V_{\max}$.

Based on the above assumptions, the trajectory of the UAV can be approximated by $U\left[n\right]=\left(x\left[n\right],y\left[n\right]\right)$, n=1,2,…N. Accordingly, the distance between the UAV and the ST, the distance from the *k*-th buoy to the UAV, and the distance from the *k*-th buoy to the ST are given by

(1) $$d^{US}\left[n\right]=\sqrt{{∥U[n]-O∥}^{2}},$$

(2) $$d_{k}^{BU}\left[n\right]=\sqrt{{∥U\left[n\right]-G_{k}∥}^{2}+H^{2}},\;k\in K,$$

(3) $$d_{k}^{BS}=\sqrt{{∥G_{k}-O∥}^{2}+H^{2}},\;k\in K,$$

where $∥•∥$ denotes the Euclidean norm of a vector.

Because of the unobstructed marine environment, buoys are rarely surrounded by large shelters, which means the communication link between the UAV, the ST, and the buoys can be regarded as LOS links [35], where the channel quality depends only on the UAV-buoy/ST distance [36]. Furthermore, the Doppler effect caused by UAV mobility is assumed to be well compensated at the receivers. Thus, the channel power gain follows the free-space path loss model that has been widely used for UAV-enabled wireless networks [25,26]. Accordingly, the channel power gain between the buoys and the UAV at time instant t∈T is

(4) $$\begin{array}{c} g_{k}^{BU}\left[n\right]=G_{0}{\left(d_{k}^{BU}\left[n\right]\right)}^{-2}=\frac{G_{0}}{{∥U\left[n\right]-G_{k}∥}^{2}+H^{2}},\;k\in K, \end{array}$$

where $G_{0}$ denotes the channel power gain at the reference distance $R_{0}$ in an LOS channel. In general, the reference distance is set as $R_{0}\;=\;1m$. Similarly, the channel power gain between the UAV and the BS is

(5) $$\begin{array}{c} g^{US}\left[n\right]=\frac{G_{0}}{{∥U[n]-O∥}^{2}}, \end{array}$$

and the channel power gain between the buoys and the ST is

(6) $$\begin{array}{c} g_{k}^{BS}\left[n\right]=\frac{G_{0}}{{∥G_{k}-O∥}^{2}+H^{2}},\;k\in K. \end{array}$$

## 3. Relay Mode Selection Mechanism and Problem Formulation

**Figure 3:**
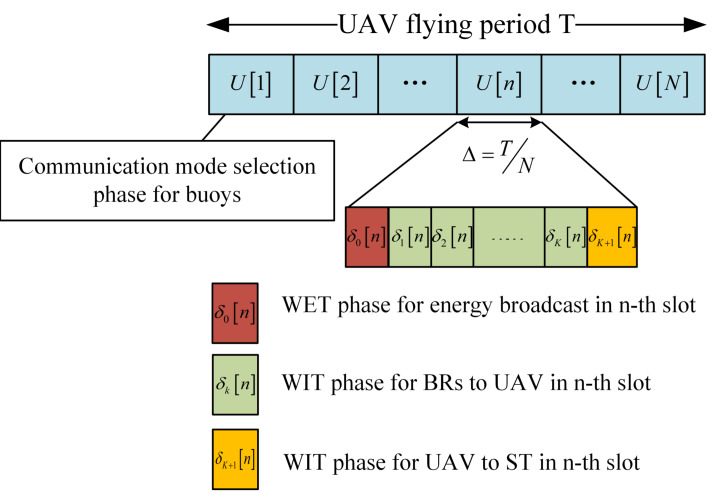
Communication protocol for the U-WPCN relay system.

In the WET phase, buoys harvest energy from the UAV, and the energy signal received by the *k*-th buoy in the *n*-th slot is

(7) $$\begin{array}{c} y_{k}\left[n\right]=s_{i}\left[n\right]\sqrt{P_{E}\left[n\right]}h_{k}^{BU}\left[n\right],\;k\in K, \end{array}$$

where $P_{E}\left[n\right]$ denotes the energy transmission power of the UAV during slot *n* and $s_{i}\left[n\right]$ is the pseudorandom energy signal with unit power. $h_{k}^{BU}\left[n\right]$ denotes the equivalent baseband channel coefficient from the UAV to the *k*-th buoy in the *n*-th slot. As UAV broadcasts a wireless energy signal to all buoys, the harvested energy of the buoys in the *n*-th slot can be expressed as

(8) $$\begin{array}{c} E_{k}\left[n\right]=\eta \delta_{0}\left[n\right]P_{E}\left[n\right]g_{k}^{BU}\left[n\right],\;k\in K, \end{array}$$

where 0<η<1 denotes the RF-to-direct current (DC) energy conversion efficiency at the energy harvester of each buoy. If the total available energy for the energy transfer at the UAV is $Q_{E}$, then $\sum_{n=1}^{N}\delta_{0}\left[n\right]P_{E}\left[n\right]\leq Q_{E}$, $0<P_{E}\left[n\right]<P_{E}^{\max}$, where $P_{E}^{\max}$ is the peak broadcast power of the UAV. In (6), $\delta_{0}\left[n\right]$ denotes the allocated subslot for the UAV energy broadcasting phase according to the time division multiple access (TDMA) protocol, as shown in Figure 3.

In the WIT phase, some of the buoys are selected for a transmission mode to complete the information transfer process.

### 3.1. Transmission Mode Selection Strategy

Assume that the buoys, UAV, and ST are working in the trusted network so that the current position and energy condition are clear for each other. In the first time slot $U\left[1\right]$, the U-WPRN system divides the buoys K into two groups, namely, buoys in direct transmission mode (BD), $K_{BD}$, and buoys in relay transmission mode (BR), $K_{BR}$, according to the current energy status, channel condition, and location of the buoys. The relationship between the BD and BR sets can be expressed as

(9) $$\begin{array}{c} K_{BD}\cup K_{BR}=K,\;K_{BD}\cap K_{BR}=\emptyset . \end{array}$$

Next, we introduce a data rate threshold $R_{thr}$. When the buoys transmit information directly to the ST at the required rate $R_{thr}$, their energy consumption for a complete communication cycle *T* will be

(10) $$\begin{array}{c} E_{k}^{DT}=\frac{\sigma^{2}}{g_{k}^{BS}}\left(2^{R_{thr}}-1\right)T,\;k\in K, \end{array}$$

the derivation of (8) is given in our previous work [23]. In this paper, after a given bandwidth *B*, $R_{thr}$ is set to the minimum value of the theoretical maximum rate $R_{k}^{\max}$ by which buoys can communicate directly with ST, i.e.,

(11) $$\begin{array}{c} R_{k}^{\max}=B\log_{2}\left(1+\frac{g_{k}^{BS}P_{k}^{\max}}{\sigma^{2}}\right), \end{array}$$

(12) $$\begin{array}{c} R_{thr}=\min\left\{R_{1}^{\max},R_{2}^{\max},\ldots ,R_{k}^{\max}\right\},\;k\in K, \end{array}$$

where $P_{k}^{\max}$ is the peak transmission power of BRs, and $\sigma^{2}$ denotes the power of the additive white Gaussian noise (AWGN) at the ST receiver. As a classic noise and interference model, AWGN is widely used in UAV-enabled LOS communication scenarios, see, for instance [11,12,13,14,15,26,27].

Based on the above considerations, the *k*-th buoy will be marked as BD in the communication cycle *T* when the initial energy reserve $B_{k}\left[0\right]$ is enough to support the consumption $E_{k}^{DT}$ in direct transmission mode. Conversely, the buoy will be marked as BR and works in the relay transmission mode.

### 3.2. Direct Transmission Mode (DT Mode)

In DT mode, the initial energy of the BDs ensures the required data rate to communicate directly with the ST; however, BDs can also harvest the energy broadcasted from the UAV in the DL. Therefore, BDs can achieve higher throughput $D_{k}^{DT}$ in the flying period *T*, concretely,

(13) $$\begin{array}{c} D_{k}^{DT}=\left\{\begin{array}{cc} TR_{thr}+\sum_{n=1}^{N}\log_{2}\left(1+\frac{g_{k}^{GB}E_{k}\left[n\right]}{\sigma^{2}\Delta }\right), & P_{k}^{ave}<P_{k}^{\max}\\ TR_{k}^{\max}, & P_{k}^{ave}=P_{k}^{\max} \end{array}\right. \end{array}$$

where $P_{k}^{ave}=\frac{E_{k}^{DT}+\sum_{n=1}^{N}E_{k}\left[n\right]}{T}$ denotes the average transmission power of the *k*-th BD over the UAV flying period *T*.

### 3.3. Relay Transmission Mode (RT Mode)

In RT mode, this paper considers a DF relay model that uses a time division duplexing (TDD) approach, and BRs can share the whole frequency allocated by the RT mode through a TDMA protocol as shown in Figure 3.

Assume that there are *K* BRs working in the RT mode with $\left|K_{BR}\right|=K$. The time slot of length Δ is further divided into K+2 subslots, and $\delta_{0}\left[n\right]+\sum_{k=1}^{K}\delta_{k}\left[n\right]+\delta_{K+1}\left[n\right]=\Delta$, which means in the *n*-th slot, the first subslot $\delta_{0}\left[n\right]$ is used for the DL WET phase. In the UL, the *k*-th BR transmits information to the UAV in subslot $\delta_{k}\left[n\right],n\in \left[2,N\right]$, $k\in K_{BR}$, and the last subslot $\delta_{K+1}\left[n\right]$ is allocated for the UAV to forward the information to the ST.

Assume that the transmitting power of the *k*-th BR in subslot $\delta_{k}\left[n\right]$ is $P_{k}\left[n\right],n\in \left[2,N\right]$ and $P_{k}\left[n\right]\leq P_{k}^{\max}$, where $P_{k}^{\max}$ denotes the peak transmission power of the BRs. Ignoring the constant energy loss of the internal circuit, the stored energy of the *k*-th BR before the subslot $\delta_{k}\left[n\right]$ can be given as

(14) $$\begin{array}{c} B_{k}\left[n\right]=\sum_{i=1}^{n}E_{k}\left[i\right]-\delta_{k}P_{k}\left[i-1\right]+B_{k}\left[0\right]. \end{array}$$

It is worth noting that when n=1, $P_{k}\left[0\right]=0$.

To achieve the self-sustainable operation of the buoys, the energy neutrality constraint is considered at each buoy *k*, which means that in the *n*-th slot, the buoy’s energy consumption for uplink information transfer cannot exceed the sum of reserve energy and energy collected from the downlink.

(15) $$\begin{array}{c} \sum_{i=2}^{n}\delta_{k}\left[i\right]P_{k}\left[i\right]\leq \sum_{i=1}^{n}E_{k}\left[i\right]+B_{k}\left[0\right]. \end{array}$$

The data rate $R_{k}$ from the *k*-th BR to the UAV in the *n*-th slot can be expressed as

(16) $$\begin{array}{c} R_{k}^{BU}\left[n\right]=\delta_{k}\left[n\right]\log_{2}\left(1+\frac{g_{k}^{BU}\left[n\right]P_{k}\left[n\right]}{\sigma^{2}}\right),n\in \left[2,N\right]. \end{array}$$

In the subslot $\delta_{K+1}$, the UAV forwards the information received from the BRs to the ST. The associated data rate from the UAV to the ST in the *n*-th slot is

(17) $$\begin{array}{c} R^{US}\left[n\right]=\delta_{K+1}\left[n\right]\log_{2}\left(1+\frac{g^{US}\left[n\right]P_{I}\left[n\right]}{\sigma^{2}}\right),n\in \left[2,N\right], \end{array}$$

where $P_{I}\left[n\right]$ denotes the information transmission power of the UAV during the *n*-th slot; $0\leq P_{I}\left[n\right]\leq P_{I}^{\max}$, where $P_{I}^{\max}$ denotes the maximum achievable transmission power for the UAV to forward the BR’s information. Clearly, the forwarded information to the ST must meet

(18) $$\begin{array}{c} \sum_{k=1}^{K}R_{k}^{BU}\left[n\right]\geq R^{US}\left[n\right], \end{array}$$

and the actual forward data rate between the BRs and the UAV in the *n*-th slot can be expressed as

(19) $$\begin{array}{c} R_{k}^{BS}\left[n\right]=\left\{\begin{array}{cc} R_{k}^{BU}\left[n\right], & \sum_{i=1}^{K}R_{i}^{BU}\left[n\right]\leq R^{US}\left[n\right]\\ R^{US}\left[n\right]\frac{R_{k}^{BU}\left[n\right]}{\sum_{i=1}^{K}R_{i}^{BU}\left[n\right]}, & otherwise. \end{array}\right. \end{array}$$

### 3.4. Problem Formulation

To ensure the fairness of BRs’ throughput in the relay mode, the objective of this paper is to maximize the minimum throughput between the BR and the ST by optimizing the UAVs trajectory $U\left[n\right]$; the subslot allocation $\delta_{0}\left[n\right]$, $\delta_{k}\left[n\right]$, and $\delta_{K+1}\left[n\right]$; the UAV’s power allocation $P_{E}\left[n\right]$ and $P_{I}\left[n\right]$; and the BR’s power allocation $P_{k}\left[n\right]$. The minimum throughput of the BRs can be expressed as

(20) $$\begin{array}{c} \begin{array}{c} D_{\min}^{BS}=\min_{k\in K_{BR}}\left\{\sum_{n=2}^{N}R_{k}^{BS}\left[n\right]\right\}\\ \;=\min_{k\in K_{BR}}\left\{\sum_{n=2}^{N}R^{US}\left[n\right]\frac{R_{k}^{BU}\left[n\right]}{\sum_{i=1}^{K}R_{i}^{BU}\left[n\right]}\right\}, \end{array} \end{array}$$

and the optimization problem can be formulated as

$$\left(P1\right)\max_{U\left[n\right],P_{E}\left[n\right],P_{I}\left[n\right],P_{k}\left[n\right],\delta_{k}}D_{\min}^{BS}$$

subject to Equations (15) and (19), as well as the following constraints,

(21) $$\begin{array}{c} \sum_{n=2}^{N}R_{k}^{BS}\left[n\right]\geq D_{\min}^{BS}, \end{array}$$

(22) $$\begin{array}{c} \sum_{k=1}^{K}\delta_{0}\left[n\right]+\delta_{k}\left[n\right]+\delta_{K+1}\left[n\right]=\Delta ,\delta_{k}\left[1\right]=0, \end{array}$$

(23) $$\begin{array}{c} \sum_{n=1}^{N}\delta_{0}\left[n\right]P_{E}\left[n\right]\leq Q_{E},0\leq P_{E}\left[n\right]\leq P_{E}^{\max}, \end{array}$$

(24) $$\begin{array}{c} 0\leq P_{I}\left[n\right]\leq P_{I}^{\max},P_{I}\left[1\right]=0, \end{array}$$

(25) $$\begin{array}{c} 0\leq P_{k}\left[n\right]\leq P_{k}^{\max},P_{k}\left[1\right]=0, \end{array}$$

(26) $$\begin{array}{c} {∥U\left[n\right]-U\left[n-1\right]∥}^{2}\leq V_{\max}\Delta , \end{array}$$

(27) $$\begin{array}{c} U\left[1\right]=U\left[N\right],k\in K_{BR},n\in \left[1,N\right], \end{array}$$

where $V_{\max}$ denotes the maximum achievable speed of the UAV.

For problem *P*1, the objective function is nonconcave and constraints (15), (19), and (21) are nonconvex due to the complicated throughput and energy functions with respect to coupled variables such as the UAV trajectory and subslot allocation scheme. According to the definition of convex optimization [37], *P*1 is a nonconvex optimization problem that is difficult to be optimally solved in general. To address this problem, we propose an effective iteration algorithm and obtain a suboptimal solution in the next section.

## 4. Proposed Algorithm

As there is no standard method for solving the nonconvex optimization problem *P*1 efficiently, in this section, we propose an iterative algorithm for *P*1 through applying the BCD [31] and SCA techniques [37]. Specifically, after dividing *P*1 into five suboptimization problems by the BCD algorithm, for a given UAV trajectory $U\left[n\right]$, UAV power allocation $P_{E}\left[n\right]$, $P_{I}\left[n\right]$, and the BR’s power control $P_{k}\left[n\right]$, we optimize the subslot allocation $\delta_{0}\left[n\right]$, $\delta_{k}\left[n\right]$ and $\delta_{K+1}\left[n\right]$ by solving a linear programming (LP) problem. For given $\delta_{0}\left[n\right]$, $\delta_{k}\left[n\right]$, $\delta_{K+1}\left[n\right]$, $U\left[n\right]$, $P_{E}\left[n\right]$, and $P_{k}\left[n\right]$, we find the closed solution for the energy-efficiency $P_{I}\left[n\right]$. The UAV’s trajectory $U\left[n\right]$, UAV power allocation $P_{E}\left[n\right]$, and the BR’s power control $P_{k}\left[n\right]$ are optimized based on the successive convex optimization technique [32]. Then, we introduce the low-complexity initialization parameters and finally give the overall algorithm. The UAV’s trajectory $U\left[n\right]$; the subslot allocation $\delta_{0}\left[n\right]$, $\delta_{k}\left[n\right]$, and $\delta_{K+1}\left[n\right]$; the UAV power allocation $P_{E}\left[n\right]$, $P_{I}\left[n\right]$; and the BR’s power control $P_{k}\left[n\right]$ are optimized in turn.

### 4.1. Subslot Allocation Optimization

For any given UAV trajectory $U\left[n\right]$ and resource allocation $P_{I}\left[n\right]$, $P_{E}\left[n\right]$, and $P_{k}\left[n\right]$, the optimal allocation problem of subslot $\delta_{0}\left[n\right]$, $\delta_{k}\left[n\right]$ and $\delta_{K+1}\left[n\right]$ in the *r*-th iteration can be given as

$$\left(P2\right)\max_{\delta_{0}\left[n\right],\delta_{k}\left[n\right],\delta_{K+1}\left[n\right]}D_{\min}^{BS}$$

s.t.(15),(19),(21),(22).

As the UAV forwards the BRs’ information based on the DF protocol, as was concluded in [19], the most effective condition is achieved when the transmission rate from the BRs to the UAV is equal to the transmission rate of the UAV to the ST, which can be expressed as

(28) $$\begin{array}{c} \sum_{k=1}^{K}R_{k}^{BU}\left[n\right]=R^{US}\left[n\right]. \end{array}$$

Under the premise of guaranteeing (28), the problem *P*2 can be rewritten equivalently as

$$\left(P2.1\right)\max_{\delta_{0}\left[n\right],\delta_{k}\left[n\right],\delta_{K+1}\left[n\right]}D_{\min}^{US}$$

subject to Equations (15), (22), and (28), as well as the following constraints,

(29) $$\begin{array}{c} \sum_{n=2}^{N}R^{US}\left[n\right]\geq D_{\min}^{US},n\in \left[2,N\right]. \end{array}$$

The objective of problem *P*2.1 is to maximize the minimum throughput between the BR and the ST. According to [38], *P*2.1 is an LP problem because the expressions in (15), (22), (28), and (29) are all affine with respect to $\delta_{k}$ and the objective function is linear. Therefore, the problem can be solved efficiently by using a commercial LP package or CVX, and the optimal subslot allocation in the *n*-th slot is denoted as $\delta_{k}^{r}=\left\{\delta_{0}^{r}\left[n\right],\delta_{k}^{r}\left[n\right],\delta_{K+1}^{r}\left[n\right]\right\},n\in \left[2,N\right],k\in \left[1,K\right]$.

### 4.2. UAV Transmission Power Optimization

As the UAV’s forwarding power is constrained by (24), the objective of UAV forwarding power optimization is to minimize the gap between $\sum_{k=1}^{K}R_{k}^{BU}\left[n\right]$ and $R^{US}\left[n\right]$ after given a UAV trajectory $U\left[n\right]$ and resource allocation $P_{E}\left[n\right]$, $P_{k}\left[n\right]$, $\delta_{0}\left[n\right]$, $\delta_{k}\left[n\right]$ and $\delta_{K+1}\left[n\right]$. Concretely,

$$\left(P3\right)\min_{P_{I}\left[n\right]}\gamma \left[n\right]$$

s.t.(24),

while

(30) $$\begin{array}{c} \gamma \left[n\right]=\sum_{k=1}^{K}R_{k}^{BU}\left[n\right]-R^{US}\left[n\right],k\in K_{BR},n\in \left[2,N\right]. \end{array}$$

The relationship between $P_{I}\left[n\right]$ and $\gamma \left[n\right]$ can be deduced from (30),

(31) $$\begin{array}{c} \begin{array}{c} \gamma \left[n\right]=\sum_{k=1}^{K}R_{k}^{BU}\left[n\right]-R^{US}\left[n\right]\\ \Rightarrow \gamma \left[n\right]=\sum_{k=1}^{K}R_{k}^{BU}\left[n\right]-\delta_{K+1}\left[n\right]\log_{2}\left(1+\frac{g^{US}\left[n\right]P_{I}\left[n\right]}{\sigma^{2}}\right)\\ \Rightarrow \frac{\sum_{k=1}^{K}R_{k}^{BU}\left[n\right]}{\delta_{K+1}\left[n\right]}-\gamma \left[n\right]=\log_{2}\left(1+\frac{g^{US}\left[n\right]P_{I}\left[n\right]}{\sigma^{2}}\right)\\ \Rightarrow 2^{\Phi \left[n\right]-\gamma \left[n\right]}-1=\frac{g^{US}\left[n\right]P_{I}\left[n\right]}{\sigma^{2}}\\ \Rightarrow P_{I}\left[n\right]=\frac{\sigma^{2}\left(2^{\Phi \left[n\right]-\gamma \left[n\right]}-1\right)}{g^{US}\left[n\right]}, \end{array} \end{array}$$

where $\Phi \left[n\right]=\frac{\sum_{k=1}^{K}R_{k}^{BU}\left[n\right]}{\delta_{K+1}\left[n\right]}$. According to Equation (31), $\gamma \left[n\right]$ decreases monotonically with the increase in $P_{I}\left[n\right]$ when $\gamma \left[n\right]=0$ and (28) is satisfied, and the most energy-efficient $P_{I}\left[n\right]$ can be expressed as

(32) $$\begin{array}{c} {\bar{P}}_{I}^{r}\left[n\right]=\frac{\sigma^{2}\left(2^{\Phi \left[n\right]}-1\right)}{g^{US}\left[n\right]} \end{array}$$

therefore, the optimal UAV transmission power in the *r*-th iteration cycle is $P_{I}^{r}=\left\{\min\left({\bar{P}}_{I}^{r}\left[n\right],P_{I}^{\max}\right)\right\}$.

### 4.3. UAV Trajectory Optimization

For any given resource allocation $\delta_{0}\left[n\right]$, $\delta_{k}\left[n\right]$, $\delta_{K+1}\left[n\right]$, $P_{E}\left[n\right]$, $P_{I}\left[n\right]$, and $P_{k}\left[n\right]$, in the *r*-th iteration, the UAV trajectory of optimal problem *P*1 can be optimized by solving the following problem,

$$\left(P4\right)\max_{U\left[n\right]}D_{\min}^{US}$$

s.t.(15),(18),(26),(27),(28).

As *P*4 is neither a concave nor a quasiconcave maximization problem due to the nonconvex constraints in (15), (18), and (29), for an optimized UAV trajectory, after introducing the slack variables $M_{k}\left[n\right]\geq {∥U\left[n\right]-G_{k}∥}^{2}+H^{2}$ and $M_{0}\left[n\right]\geq {∥U[n]-O∥}^{2}$, the optimization problem *P*4 can be reformulated as

$$\left(P4.1\right)\max_{U\left[n\right],M_{k}\left[n\right],M_{0}\left[n\right]}D_{\min}^{US}$$

subject to Equations (26) and (28), as well as the following constraints,

(33) $$\begin{array}{c} \sum_{n=2}^{N}\delta_{k}\left[n\right]P_{k}\left[n\right]\leq B_{k}\left[0\right]+\sum_{i=1}^{n}\frac{G_{0}\eta \delta_{0}\left[n\right]P_{E}\left[i\right]}{M_{k}\left[i\right]}, \end{array}$$

(34) $$\begin{array}{c} \sum_{n=2}^{N}\delta_{K+1}\left[n\right]\log_{2}\left(1+\frac{\frac{G_{0}}{\sigma^{2}}P_{I}\left[n\right]}{M_{0}\left[n\right]}\right)\geq D_{\min}^{US}, \end{array}$$

(35) $$\begin{array}{c} \begin{array}{c} \sum_{k=1}^{K}\delta_{k}\left[n\right]\log_{2}\left(1+\frac{\frac{G_{0}}{\sigma^{2}}P_{k}\left[n\right]}{M_{k}\left[n\right]}\right)\geq \delta_{K+1}\left[n\right]\log_{2}\left(1+\frac{\frac{G_{0}}{\sigma^{2}}P_{I}\left[n\right]}{M_{0}\left[n\right]}\right), \end{array} \end{array}$$

(36) $$\begin{array}{c} M_{k}\left[n\right]\geq {∥U\left[n\right]-G_{k}∥}^{2}+H^{2}, \end{array}$$

(37) $$\begin{array}{c} M_{0}\left[n\right]\geq {∥U[n]-O∥}^{2},k\in K_{BR},n\in \left[1,N\right]. \end{array}$$

Notably, as constraints (36) and (37) must be satisfied to obtain the optimal solution of *P*4.1, we can enlarge the upper bound of the objective value $D_{\min}^{US}$ as shown in (34) by decreasing $M_{0}\left[n\right]$, such that *P*4.1 is equivalent to *P*4.

The convex function is lower-bounded by its first-order Taylor expansion at any point [37]; thus, with the given trajectory of the UAV in the (r−1)-th iteration, $M_{k}^{r-1}\left[n\right]$ represents the square of the distance between the UAV and the BRs in the (r−1)-th iteration. Then, at local point U[n], the lower bound of $R_{k}^{BU}\left[n\right]$ is

(38) $$\begin{array}{c} \begin{array}{c} R_{k}^{BU}\left[n\right]=\delta_{k}\left[n\right]\log_{2}\left(1+\frac{\frac{G_{0}}{\sigma^{2}}P_{k}\left[n\right]}{M_{k}\left[n\right]}\right)\\ \;\geq {\tilde{R}}_{k}^{BU}\;=\delta_{k}\left[n\right]\left[\alpha_{k}^{r-1}\left[n\right]-\beta_{k}^{r-1}\left[n\right]\left(M_{k}\left[n\right]-M_{k}^{r-1}\left[n\right]\right)\right]\;, \end{array} \end{array}$$

where

(39) $$\begin{array}{c} \alpha_{k}^{r-1}\left[n\right]=\log_{2}\left(1+\frac{\frac{G_{0}}{\sigma^{2}}P_{k}\left[n\right]}{M_{k}^{r-1}\left[n\right]}\right), \end{array}$$

(40) $$\begin{array}{c} \beta_{k}^{r-1}\left[n\right]=\frac{\frac{G_{0}}{\sigma^{2}\ln2}P_{k}\left[n\right]}{\left(M_{k}^{r}\left[n\right]+\frac{G_{0}}{\sigma^{2}}P_{k}\left[n\right]\right)M_{k}^{r-1}\left[n\right]}. \end{array}$$

Similarly, $M_{0}^{r-1}\left[n\right]$ represents the square of the distance between the UAV and the ST in the *r*-th iteration, and $R^{US}\left[n\right]$ is lower-bounded by

(41) $$\begin{array}{c} \begin{array}{c} R^{US}\left[n\right]=\delta_{K+1}\left[n\right]\log_{2}\left(1+\frac{\frac{G_{0}}{\sigma^{2}}P_{I}\left[n\right]}{M_{0}\left[n\right]}\right)\\ \;\geq {\tilde{R}}^{US}\;=\delta_{K+1}\left[n\right]\left[\alpha_{B}^{r-1}\left[n\right]\;-\beta_{B}^{r-1}\left[n\right]\left(M_{0}\left[n\right]\;-M_{0}^{r-1}\left[n\right]\right)\right]\;, \end{array} \end{array}$$

where

(42) $$\begin{array}{c} \alpha_{B}^{r-1}\left[n\right]=\log_{2}\left(1+\frac{\frac{G_{0}}{\sigma^{2}}P_{I}\left[n\right]}{M_{0}^{r-1}\left[n\right]}\right), \end{array}$$

(43) $$\begin{array}{c} \beta_{B}^{r-1}\left[n\right]=\frac{\frac{G_{0}}{\sigma^{2}\ln2}P_{I}\left[n\right]}{\left(M_{0}^{r}\left[n\right]+\frac{G_{0}}{\sigma^{2}}P_{I}\left[n\right]\right)\left(M_{0}^{r-1}\left[n\right]\right)}. \end{array}$$

Next, the lower bound of $E_{k}\left[n\right]$ can be obtained through a first-order Taylor expansion,

(44) $$\begin{array}{c} \begin{array}{c} E_{k}\left[n\right]=\frac{\eta \delta_{0}\left[n\right]G_{0}P_{E}\left[n\right]}{{∥U\left[n\right]-G_{k}∥}^{2}+H^{2}}\\ \geq {\tilde{E}}_{k}\left[n\right]=\frac{\eta \delta_{0}\left[n\right]G_{0}P_{E}\left[n\right]}{M_{k}^{r-1}\left[n\right]}-\frac{\eta \delta_{0}\left[n\right]G_{0}P_{E}\left[n\right]\left(M_{k}\left[n\right]-M_{k}^{r-1}\left[n\right]\right)}{{\left(M_{k}^{r-1}\left[n\right]\right)}^{2}}. \end{array} \end{array}$$

Based on the above considerations, problem *P*4.1 can be approximated as

$$\left(P4.2\right)\max_{U\left[n\right],M_{k}\left[n\right],M_{0}\left[n\right]}D_{\min}^{US}$$

subject to Equations (26) and (27), as well as the following constraints.

(45) $$\begin{array}{c} \sum_{n=2}^{N}{\tilde{R}}^{US}\left[n\right]\geq D_{\min}^{US}, \end{array}$$

(46) $$\begin{array}{c} \sum_{n=2}^{N}\delta_{k}\left[n\right]P_{k}\left[n\right]\leq B_{k}\left[0\right]+\sum_{i=1}^{n}{\tilde{E}}_{k}\left[i\right], \end{array}$$

(47) $$\begin{array}{c} \sum_{k=1}^{K}{\tilde{R}}_{k}^{BU}\left[n\right]\geq {\tilde{R}}^{US}\left[n\right],k\in K_{BR},n\in \left[1,N\right]. \end{array}$$

*P*4.2 is a convex optimization problem that can be efficiently solved by standard convex optimization solvers such as CVX [39]. In addition, as the constraints in *P*4.2 are lower bounds compared with *P*4.1, any feasible solution of *P*4.2 is also feasible for *P*4.1, but the reverse does not hold in general. Finally, by solving *P*4.2, the optimal UAV trajectory $U^{r}=\left\{U^{r}\left[n\right]\right\},n\in \left[1,N\right]$ can be obtained to maximize the minimum collecting data rate from the BRs.

### 4.4. UAV Broadcast Power Optimization

For any given UAV trajectory $U\left[n\right]$ and resource allocation $P_{I}\left[n\right]$, $P_{k}\left[n\right]$, and $\delta_{k}$, the optimization problem of UAV energy broadcast power can be rewritten as

$$\left(P5\right)\max_{P_{E}\left[n\right]}D_{\min}^{BU}$$

s.t.(15),(23),(29).

It is clear that the target of UAV broadcast power optimization is to maximize the minimum energy that the BR collects during the UAV flight period *T* while ensuring the energy consumption constraint (15) to further maximize $D_{\min}^{BU}$. The reason is that the transmission power of the BR is largely determined by the current energy situation, and the energy broadcast by the drone is an important source of energy for the BR. In addition, maintaining (15) can guarantee the convergence of the BCD method. The optimization problem can be reformulated as

$$\left(P5.1\right)\max_{P_{E}\left[n\right]}E_{k}^{\min}$$

subject to Equations (15) and (23), as well as the following constraints,

(48) $$\begin{array}{c} E_{k}=\sum_{n=1}^{N}\eta \delta_{0}\left[n\right]P_{E}\left[n\right]g_{k}^{BU}\left[n\right]\geq E_{k}^{\min}. \end{array}$$

In problem *P*5.1, the slack variable $E_{k}^{\min}$ is introduced to denote the minimum harvested energy of the BRs; in addition, $E_{k}\geq E_{k}^{\min}$, and $E_{k}$ is the sum of the received energy of the *k*-th BR. Clearly, *P*5.1 is a typical convex optimization problem and thus can be solved efficiently by CVX. In the *n*-th slot, the optimal energy broadcast power of the UAV is denoted as $P_{E}^{r+1}=\left\{P_{E}^{r}\left[n\right]\right\},n\in \left[1,N\right]$.

### 4.5. BR Transmission Power Optimization

In the *r*-th iteration, with a given UAV trajectory U[n] and the resource allocation of $\delta_{0}\left[n\right]$, $\delta_{k}\left[n\right]$, $\delta_{K+1}\left[n\right]$, $P_{E}\left[n\right]$, and $P_{I}\left[n\right]$, the BR’s information transmission power in the *n*-th slot can be optimized by

$$\left(P6\right)\max_{P_{k}\left[n\right]}D_{\min}^{BU}$$

subject to Equations (15) and (25), as well as the following constraints,

(49) $$\begin{array}{c} \sum_{n=2}^{N}R^{BU}\left[n\right]\geq D_{\min}^{BU},n\in \left[2,N\right]. \end{array}$$

Due to the nonconvex constraint (49), problem *P*6 is a nonconvex optimization problem, and we can perform first-order Taylor expansion of (49) in terms of $P_{k}$, which can be expressed as

(50) $$\begin{array}{c} \begin{array}{c} R_{k}^{BU}\left[n\right]=\delta_{k}\left[n\right]\log_{2}\left(1+\frac{g^{BU}\left[n\right]P_{k}\left[n\right]}{\sigma^{2}}\right)\\ \begin{array}{cc} \geq & \delta_{k}\left[n\right]\log_{2}\left(1+\frac{g^{BU}\left[n\right]P_{k}^{r}\left[n\right]}{\sigma^{2}}\right) \end{array}+\frac{\delta_{k}\left[n\right]}{\ln2\left(P_{k}^{r}\left[n\right]+\frac{\sigma^{2}}{g^{BU}\left[n\right]}\right)}\left(P_{k}\left[n\right]-P_{k}^{r}\left[n\right]\right) \end{array}. \end{array}$$

After substituting (50) into formula (49), *P*6 becomes a typical convex optimization problem that can be solved efficiently by optimization tools such as CVX. In the *n*-th slot, the optimal information transmission power of the *k*-th BR is denoted as $P_{k}^{r}=\left\{P_{k}^{r}\left[n\right]\right\},n\in \left[2,N\right],k\in \left[1,K\right]$.

### 4.6. Proposed Optimization Algorithm

The proposed algorithm for problem *P*1 is given in Algorithm 1, and the initialization parameters are set as follows, the initial trajectory $U^{0}$ of the UAV is a circle with a center of ST and radius of $\frac{V_{\max}T}{4\pi }$. The initial transmission and broadcast power of the UAV are $P_{I}^{0}\left[1\right]=0$,$P_{I}^{0}\left[n\right]=\frac{1}{2}P_{I}^{\max},n\in \left[2,N\right]$, and $P_{E}^{0}\left[n\right]=\min\left(\frac{Q_{E}}{T},P_{E}^{\max}\right),n\in \left[1,N\right]$, respectively. The initial transmission power of the *k*-th buoy is $P_{k}^{0}\left[1\right]=0$, $P_{k}^{0}\left[n\right]=\min\left(E_{k}\left[n\right]+\frac{B_{k}\left[0\right]}{N-1},P_{k}^{\max}\right)$, $n\in \left[2,N\right]$, k∈K.

In the BCD method, the optimization variables in the original problem *P*1 are partitioned into five blocks, and the optimization order affects not only the convergence speed but also the robustness of the algorithm [38]. In this paper, we first optimize the time slot resource allocation scheme $\delta_{k}\left[n\right]$ for the following reason; in the block of subslot allocation optimization, the objective function is linear and the constraints (15), (22), (28), and (29) are affine with $\delta_{k}^{r}$, and there is no strong constraint; as a comparison, the trajectory is constrained by the flight speed and the power distribution is subject to the total energy and a maximum power constraint. Therefore, the time slot resource allocation is flexible compared to other optimization targets.

Notably, unlike the classic BCD method, which requires the exact optimal solution of the subproblems in each block for global optimization [38], the SCA-enabled BCD allows nonconvex subproblems to be approximated as convex problems: the approximate subproblems update each block of variables with a near-optimal solution. Moreover, the SCA-enabled BCD can be guaranteed to converge to a suboptimal solution when the objective value of *P*1 with the solutions obtained by solving the subproblem with proper optimization sequence is nondecreasing over iterations and the optimal value of P1 must be finite [15,40]. According to the above theories, the convergence of the algorithm proposed in this paper can be proved as follows.

First, in iteration $r\left(r\geq 1\right)$ of Algorithm 1, define $\phi \left(\delta_{k}^{r},P_{I}^{r},U^{r},P_{E}^{r},P_{k}^{r}\right)$ as the objective value of problem *P*1, and $\phi \left(\delta_{k}^{r+1},P_{I}^{r},U^{r},P_{E}^{r},P_{k}^{r}\right)$ as the objective value of subproblem *P*2.1. We have the following inequality,

(51) $$\begin{array}{c} \phi \left(\delta_{k}^{r},P_{I}^{r},U^{r},P_{E}^{r},P_{k}^{r}\right)\leq \phi \left(\delta_{k}^{r+1},P_{I}^{r},U^{r},P_{E}^{r},P_{k}^{r}\right), \end{array}$$

because $\delta_{k}^{r+1}$ is the optimal solution to problem *P*2.1. Second, as $\delta_{k}^{r+1}$ can maintain the information forwarding constraint (28) under $P_{I}^{r}$, for given $\delta_{k}^{r+1},P_{I}^{r},U^{r},P_{E}^{r},P_{k}^{r}$ in step 4, we have

(52) $$\begin{array}{c} \phi \left(\delta_{k}^{r+1},P_{I}^{r},U^{r},P_{E}^{r},P_{k}^{r}\right)\leq \phi \left(\delta_{k}^{r+1},P_{I}^{r+1},U^{r},P_{E}^{r},P_{k}^{r}\right), \end{array}$$

because $P_{I}^{r+1}$ is the most energy-efficient UAV forwarding power. Third, for given $\delta_{k}^{r+1},P_{I}^{r+1},U^{r},P_{E}^{r},P_{k}^{r}$ in step 5, it follows that

(53) $$\begin{array}{c} \begin{array}{ccc} \phi \left(\delta_{k}^{r+1},P_{I}^{r+1},U^{r},P_{E}^{r},P_{k}^{r}\right) & \overset{\left(a\right)}{=} & \phi_{lb}\left(\delta_{k}^{r+1},P_{I}^{r+1},U^{r},P_{E}^{r},P_{k}^{r}\right)\\ & \overset{\left(b\right)}{\leq } & \phi_{lb}\left(\delta_{k}^{r+1},P_{I}^{r+1},U^{r+1},P_{E}^{r},P_{k}^{r}\right)\\ & \overset{\left(c\right)}{\leq } & \phi \left(\delta_{k}^{r+1},P_{I}^{r+1},U^{r+1},P_{E}^{r},P_{k}^{r}\right), \end{array} \end{array}$$

similar to the nondecreasing nature of SCA in UAV trajectory demonstrated in [13,37], (53a) holds because the first-order Taylor expansions in (38), (41), and (44) are tight at the given local points, respectively; (53b) holds because $U^{r+1}$ is the near-optimal solution for *P*4.2; and (53c) holds because the objective value of *P*4.2 is the lower bound of that of its original problem *P*4 at $U^{r+1}$. In addition, for given $\delta_{k}^{r+1},P_{I}^{r+1},U^{r+1},P_{E}^{r},P_{k}^{r}$ in step 6, it follows that

(54) $$\begin{array}{c} \phi \left(\delta_{k}^{r+1},P_{I}^{r+1},U^{r+1},P_{E}^{r},P_{k}^{r}\right)=\phi \left(\delta_{k}^{r+1},P_{I}^{r+1},U^{r+1},P_{E}^{r+1},P_{k}^{r}\right), \end{array}$$

as $P_{E}^{r+1}$ can ensures the energy consumption constraint (15), it will not affect the current results immediately as shown in (55), but it can prepare for the next optimization of $P_{k}^{r}$. Given $\delta_{k}^{r+1},P_{I}^{r+1},U^{r+1},P_{E}^{r+1},P_{k}^{r}$, we have

(55) $$\begin{array}{c} \begin{array}{ccc} \phi \left(\delta_{k}^{r+1},P_{I}^{r+1},U^{r+1},P_{E}^{r+1},P_{k}^{r}\right) & \overset{\left(a\right)}{=} & \phi_{lb}\left(\delta_{k}^{r+1},P_{I}^{r+1},U^{r+1},P_{E}^{r+1},P_{k}^{r}\right)\\ & \overset{\left(b\right)}{\leq } & \phi_{lb}\left(\delta_{k}^{r+1},P_{I}^{r+1},U^{r+1},P_{E}^{r+1},P_{k}^{r+1}\right)\\ & \overset{\left(c\right)}{\leq } & \phi \left(\delta_{k}^{r+1},P_{I}^{r+1},U^{r+1},P_{E}^{r+1},P_{k}^{r+1}\right), \end{array} \end{array}$$

which can be similarly shown as in (53). Based on (51)–(55), we finally obtain

(56) $$\begin{array}{c} \phi \left(\delta_{k}^{r},P_{I}^{r},U^{r},P_{E}^{r},P_{k}^{r}\right)=\phi \left(\delta_{k}^{r+1},P_{I}^{r+1},U^{r+1},P_{E}^{r+1},P_{k}^{r+1}\right), \end{array}$$

which means that the objective value of problem P1 is nondecreasing over iterations in Algorithm 1, and a suboptimal solution can be obtained through iteration.

Based on the above analysis, the subslot allocation scheme, the UAV transmission power, the BR transmission power, the UAV trajectory, and the UAV broadcast power are alternately optimized by solving problems *P*2.1, *P*3, *P*4.2, *P*5.1, and *P*6, while keeping the other four blocks of variables fixed. Finally, a feasible suboptimal solution of the original problem *P*1 can be obtained.
**Algorithm 1** Successive Convex Optimization Algorithm1:Initialize $P_{I}^{r}$, $U^{r}$, $P_{E}^{r}$ and $P_{k}^{r}$, let r=1.2:**repeat**3: Solve problem *P*2.1 for given $\left\{P_{I}^{r},U^{r},P_{E}^{r},P_{k}^{r}\right\}$ and obtain the optimal solution as $\delta_{k}^{r+1}$.4: Solve problem *P*3 for given $\left\{\delta_{k}^{r+1},P_{I}^{r},U^{r},P_{E}^{r},P_{k}^{r}\right\}$ and obtain the optimal solution as $P_{I}^{r+1}$.5: Solve problem *P*4.2 for given $\left\{\delta_{k}^{r+1},P_{I}^{r+1},U^{r},P_{E}^{r},P_{k}^{r}\right\}$ and obtain the optimal solution as $U^{r+1}$.6: Solve problem *P*5.1 for given $\left\{\delta_{k}^{r+1},P_{I}^{r+1},U^{r+1},P_{E}^{r},P_{k}^{r}\right\}$ and obtain the optimal solution as $P_{E}^{r+1}$.7: Solve problem *P*6 for given $\left\{\delta_{k}^{r+1},P_{I}^{r+1},U^{r+1},P_{E}^{r+1},P_{k}^{r}\right\}$ and obtain the optimal solution as $P_{k}^{r+1}$.8:**until** The fluctuation of the objective value is below a threshold $0\leq D_{\min}^{BU}\left(r+1\right)-D_{\min}^{BU}\left(r\right)\leq \varepsilon ,\varepsilon >0$.

## 5. Numerical Results

In this section, numerical results are provided to evaluate the performance of the proposed algorithm. For the convenience of demonstration, consider a system with $\left|K\right|=\;9$ ground terminals that are randomly and uniformly distributed within 160,000 square meters, and the following results are obtained from one random case of the buoy’s location.

The parameter settings in this article are as follows; the peak energy broadcast power of the UAV is $P_{E}^{\max}=30\;W$, and the available energy of the UAV in the WET phase for each flying period is $Q_{E}=1\;\mathrm{Wh}$. The peak forwarding power of the UAV is $P_{I}^{\max}=1\;\mathrm{mW}$. The peak transmit power of the buoys in the WIT phase is set as $P_{k}^{\max}=0.5\;\mathrm{mW}$, and Equation (12) can be used to obtain $R_{thr}=0.012\;\mathrm{bps}/\mathrm{Hz}$. The energy harvesting efficiency of all buoys is η=0.55, and the length of the time slot is Δ=1s. The receiver noise power is assumed to be $\sigma^{2}=-90\;\mathrm{dBm}$. The communication bandwidth is B=1MHz. The channel power gain at the reference distance $R_{0}=1\;m$ of the LOS channel is set as $G_{0}=$−30 dB. Finally, the UAV flight altitude is fixed to H=10m, and the maximum speed is $V_{\max}=5\;m/s$. We selected the above simulation parameters to verify the performance of the algorithm proposed in this paper for the following reasons.

First, there are few obstructions between surface buoys and the UAV except for islands, reefs, or ships. Therefore, without losing generality, the LOS channel between surface buoys and the UAV can adopt the parameters that have been widely adopted in related papers [22,23,24,25,26,27,28,29].

Second, the flying height and speed of the UAV usually depend on the height of obstacles and the level of wind speed [41]. As there are few obstacles on the sea surface and the sea wind in high altitude is stronger, the numerical simulation assumes a flight speed of 5 m/s and a flight height of 10 m, so as to ensure the safe flight of the UAV while efficiently transmitting energy to the buoys [7,15].

Finally, in the U-WPRN proposed in this paper, the energy broadcast node and energy collection module produced by Powercast are installed on the UAV and buoys, respectively [42]. The UAV provides energy broadcasting and information relay services to the buoys in the coverage area periodically, and landing on the ship for charging in spare time. As the lifetime of the UAV depends on the capacity of the battery, and the load capacity of the UAV is usually a few kilograms, the cruising time of the UAV is about half an hour to two hours after installing the battery and equipment [41]. Therefore, to approach the actual situation and considering the loss of free space, the UAV coverage of 160,000 square meters is selected in numerical simulation.

### 5.1. Simulation Results for the Case $B_{k}\left[0\right]=0$

Assuming that all buoys do not have an initial energy reserve, $B_{k}\left[0\right]=\;0$ and k∈K, according to the communication mode selection strategy proposed in this paper; these buoys are marked as BRs and work in relay transmission mode $k\in K_{BR}$, $\left|K_{BR}\right|=9$.

**Figure 4:**
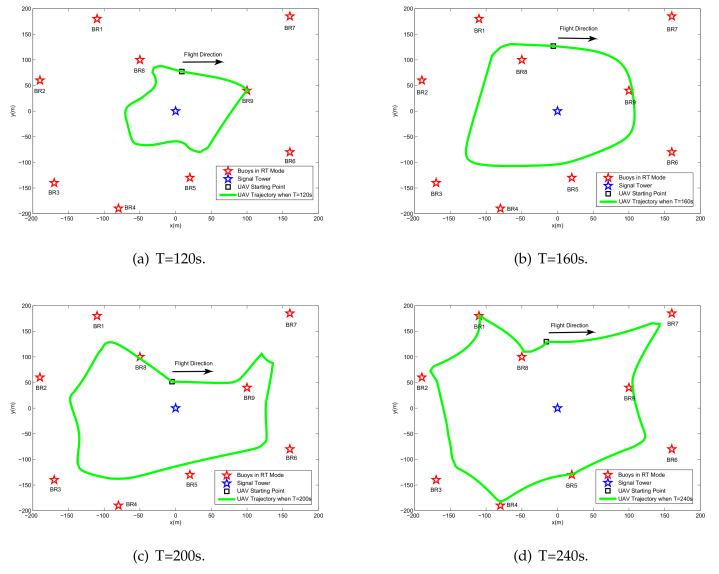
The optimized UAV trajectory for different values of period *T* for the case $B_{k}\left[0\right]=\;0$.

Figure 4 shows the UAV trajectory for different periods *T*. It is observed that when increasing the period *T*, the UAV tends to traverse each BR to perform more energy broadcasts and more information reception. The reason for this phenomenon is that due to the effect of the double near-far problem, allocating more energy and time slot resources to the BR closer to the UAV can lead to better performance. To ensure fair throughput of each BR in the flight period, by controlling the UAV energy broadcast power, BR information transmission power, UAV information forwarding power, and TDMA time slot allocation, the influence of distance is eliminated, and finally an optimized flight trajectory is obtained.

**Figure 5:**
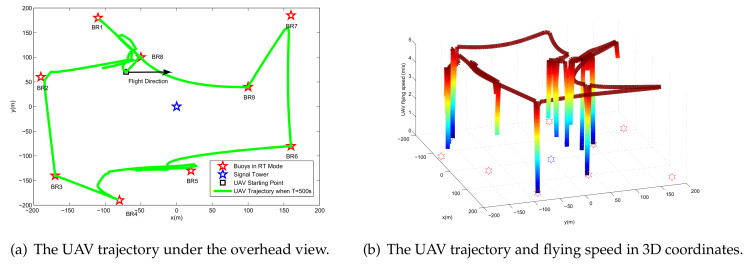
The optimized UAV trajectory and flying speed when *T* = 500 s, $B_{k}\left[0\right]=\;0$.

When the flight period is T=500 s, Figure 5a shows the UAV trajectory and Figure 5b gives the flying speed. It can be clearly seen that when the UAV passes over the BR, it tends to reduce the flight speed to better serve the BR, while in the BR-intensive area, the UAV will intermittently reduce the flight speed, thereby improving the service quality of the area. Notably, when the UAV passes over the BR7, there is no deceleration because the BR7 is farther from the other BRs. At this time, the UAV will choose to fly quickly and compensate the BR7 in other ways (e.g., improve the UAV broadcast power $P_{E}\left[n\right]$ and forward power $P_{I}\left[n\right]$ and allocate more subslot length $\delta_{0}\left[n\right],\delta_{7}\left[n\right]$) to achieve the optimization goal of this paper; that is, the overall minimum throughput is maximized.

**Figure 6:**
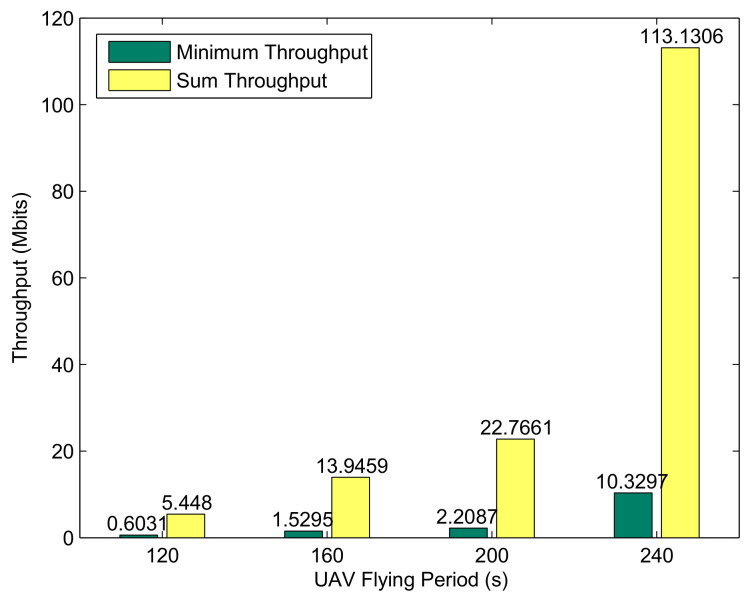
Performance comparison of different periods *T* for the case $B_{k}\left[0\right]=\;0$.

Figure 6 shows the minimum achievable throughput of the BRs and the UAV forward throughput under different periods for the case of $B_{k}\left[0\right]=0$. It is clear from the above figure that the system performance increases with the flight period, especially when T=240s. The reason for this phenomenon can be obtained by analyzing the trajectory, as shown in Figure 4. In the four preset flying periods, the UAV can traverse most buoys when T=240 s; as a comparison, only a few buoys are traversed. In addition, due to the characteristics of U-WPRN, the ability of BR to collect energy and transmit information mainly depends on the LOS channel; specifically, it depends on the distance between UAV and BR, which means that the larger the period *T* is, the closer the UAV can be to the BR. Therefore, with the increase in the flight cycle *T*, the system performance presents nonlinear growth.

### 5.2. Simulation Results for the Case $B_{k}\left[0\right]>\;0$

Normally, the buoys in the U-WPCN system have nonzero stored energy before transmission, $B_{k}\left[0\right]>0$ and k∈K. Thus, after using the WIT-phase buoy communication mode selection strategy, the buoys are assigned to two communication modes during the WIT phase and we have $\left|K_{BR}\right|=6$. For a clearer representation, the initial energy $B_{k}\left[0\right]$ of the buoys is expressed as a form associated with the energy threshold $E_{k}^{DT}$, which was given in Equation (8). In the simulation of this chapter, the parameter is $\left\{BR_{j}\right\}=\left\{0.1,0.2,0.3,0.7,0.4,0.5\right\}*E_{j}^{DT}$ and $\left\{BD_{i}\right\}\geq E_{i}^{DT}$.

**Figure 7:**
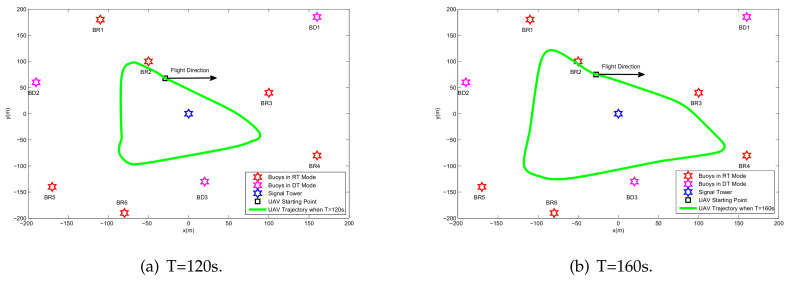

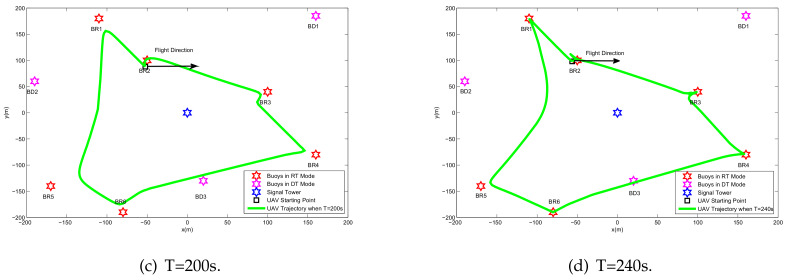
The UAV trajectories of different periods *T* for the case $B_{k}\left[0\right]>0$.

**Figure 8:**
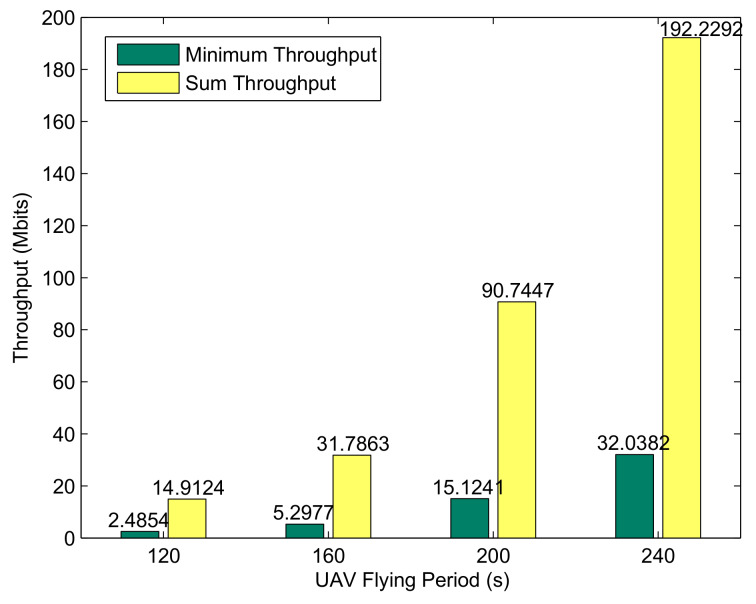
Performance comparison of different periods *T* for the case $B_{k}\left[0\right]>\;0$.

Based on the above conditions, Figure 7 shows the optimized trajectory obtained by the proposed algorithm under different periods. The UAV prioritizes the relay service for the BRs based on the energy reserve and the location relationship of the BR. In Figure 4, the UAV provides fair service for all buoys; however, in Figure 7, the UAV neither provides WIT-phase relay services for energy-rich BDs nor changes trajectories and power allocations for the BDs during the WET phase. Therefore, in Figure 8, benefiting from the pertinence of the UAV resource allocation, the buoy selection mechanism steadily improves the performance of the BRs with an increase in the flight period.

**Figure 9:**
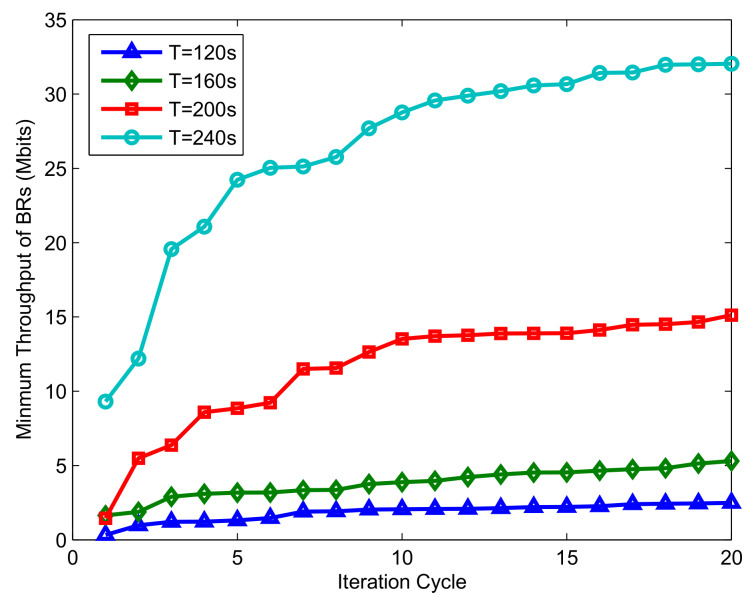
Convergence comparison of the BRs’ minimum throughput under different iterations for the case $B_{k}\left[0\right]>\;0$.

Figure 9 shows the convergence performance of the proposed algorithm. Clearly, the convergence rate is proportional to the flight period *T*, and there is always a difference in throughput in different periods. The reason is that if the fixed sampling interval is 1*s*, the larger the flight period is, the greater the number of time slots that need to be optimized; similarly, an increase in computational complexity means that more resources can be utilized and optimized, which can result in better performance. In addition, when the iteration period is large enough, the algorithm will eventually stabilize, and the resource allocation scheme with optimal system throughput can be obtained.

**Figure 10:**
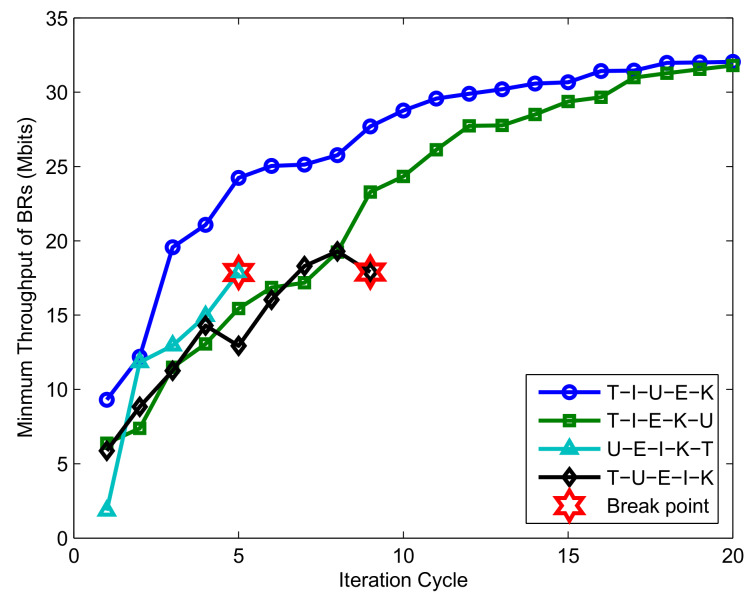
Convergence performance comparison of different optimization orders for the case $B_{k}\left[0\right]>\;0,T\;=\;$240 s.

Figure 10 shows the convergence performance of the successive convex optimization algorithm under different optimization orders. As the algorithm proposed in this paper contains five subproblems, there are theoretically 120 different optimization sequences, and Figure 10 selects four representative sequences for comparison. T, I, K, U, and E represent the optimization sequence of the time slot allocation, the UAV transmission power, the BR’s transmission power, the UAV trajectory, and the UAV broadcast power, respectively. As shown in Figure 10, when the time slot resource is optimized first and the UAV trajectory optimization is placed at the end, the algorithm can approach convergence, but the convergence speed is not quick enough. As a comparison, optimizing the UAV broadcast power after optimizing the trajectory can improve the convergence speed. The reason is that the UAV broadcast power optimization problem is designed to maximize the minimum energy collected by the BRs, which is affected by the current UAV trajectory and the time slot allocation scheme. Thus, optimal E after T and U can achieve better performance in each iteration cycle. In addition, as the trajectory optimization is strictly limited by the flying speed of the UAV, the solution space is more limited than the time slot optimization. Furthermore, the optimization T is constrained by (28); (28) is a strict equality constraint, which poses a substantial challenge to the problem of optimizing T. Based on the above consideration, if U is performed immediately after T or U is placed before T, errors may easily occur.

**Figure 11:**
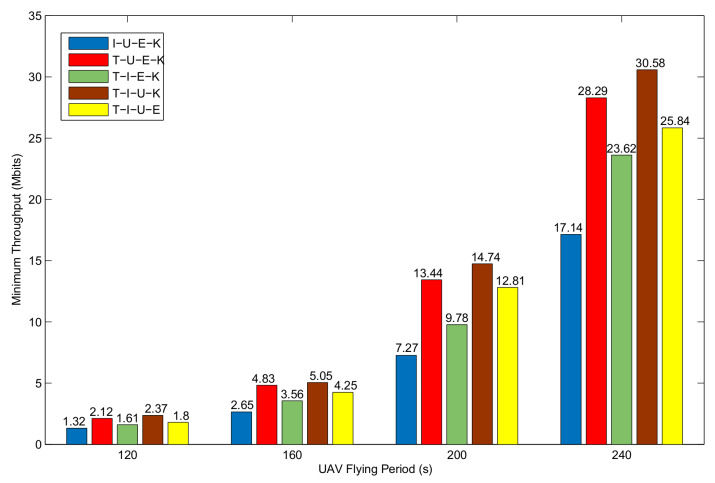
Performance comparison of different optimization target when $B_{k}\left[0\right]>0$.

Figure 11 shows the performance comparison when evaluating algorithms proposed in this paper to optimize four of the five potential targets. Notably, the worse the optimization result is, the more important the variable that represents no optimization. It can be seen that when optimizing the remaining four items and ignoring subslot optimization, the system performance is the worst. The reason is that the subslot allocation scheme has a great impact on system performance due to the TDMA protocal. In addition, as the LOS channel conditions depend on the distance, optimizing the UAV trajectory can significantly improve system performance.

### 5.3. Comparison of Optimization Results between $B_{k}\left[0\right]=0$ and $B_{k}\left[0\right]>0$

**Figure 12:**
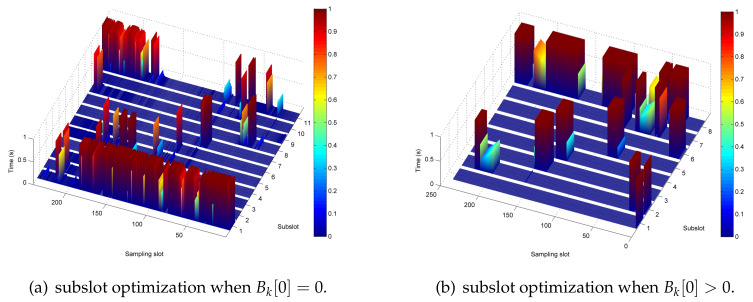

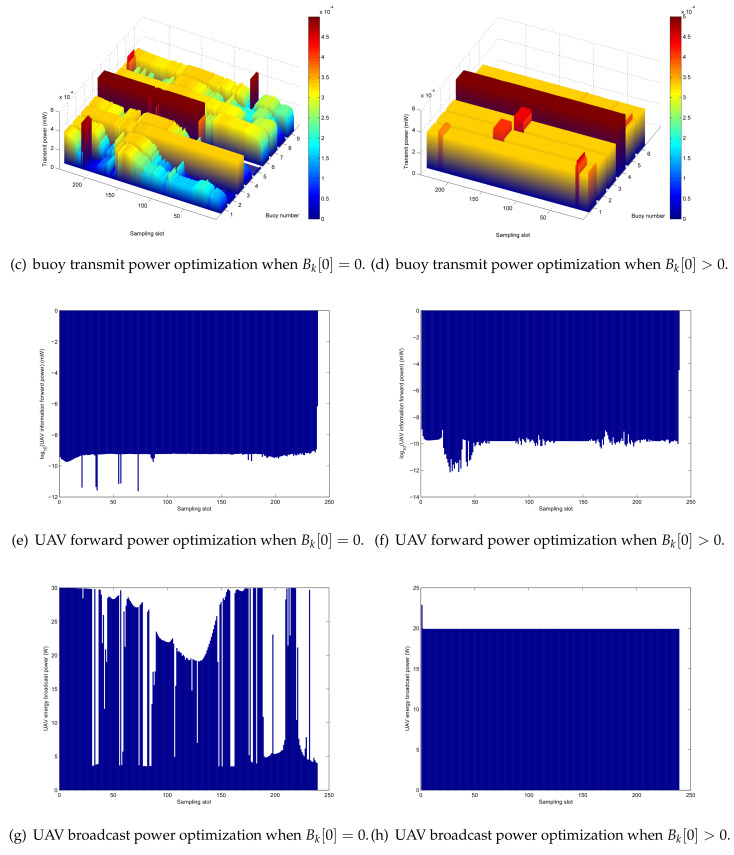
Optimization results of $B_{k}\left[0\right]=0$ and $B_{k}\left[0\right]>0$ when *T* = 240 s.

Figure 12 shows the optimization results of the system in the case of $B_{k}\left[0\right]=0$ and $B_{k}\left[0\right]>0$ when T=240 s. First, by comparing the allocation of sub-timeslot resources in Figure 12a,b, it can be seen that when $B_{k}\left[0\right]=0$, the time slots are mainly used for UAV energy broadcasting; however, when $B_{k}\left[0\right]>0$, certain time slots are used more for UAV information forwarding. Then, Figure 12c,d shows the information transmission power allocation optimization schemes for buoys in different energy conditions. When $B_{k}\left[0\right]=0$, there is a significant fluctuation in the power allocation of the buoy due to the energy harvesting situation. As a comparison, when $B_{k}\left[0\right]>0$, the information transmission power allocation of the BR is relatively stable. Furthermore, for ease of description, the UAV information forward power after logarithmic transformation is given in Figure 12e and Figure 12f, respectively. Finally, the UAV broadcast power optimization results are given in Figure 12g,h. Obviously, when $B_{k}\left[0\right]=0$, the closer the UAV is to the buoy, the higher is the energy broadcast, and when $B_{k}\left[0\right]>0$, the UAV energy broadcast power is higher at the first sampling instance and subsequently remains in a stable state.

## 6. Conclusions and Future Work

In this paper, a U-WPRN with a buoy communication mode selection strategy has been investigated, in which the UAV acts as a flight power station and an information relay node and improves service for energy-poor ocean buoy networks. The UAV trajectory, the UAV broadcast and transmission power, the buoys’ transmission power and the time slot allocation in the TDMA protocol are jointly optimized to maximize the minimum throughput for energy-short buoys. To solve the nonconvex problem, the original optimization problem is divided into five subproblems and solved by alternating optimization and successive convex optimization techniques. Due to the convergence of the algorithm, a feasible suboptimal solution can be efficiently obtained by the proposed strategy. Numerical results demonstrate the efficiency of the proposed algorithm in different scenarios.

In the study of U-WPRN construction, the aim of this paper was to maximize the minimum throughput of the buoys during the flight period and to further mitigate the effects of the double near-far phenomenon and energy shortage for the U-WPRN. However, compared with hovering UAVs, due to the cyclical tendency of resource allocation, flying UAVs cannot guarantee the real-time communication data rate of the buoys, and hovering UAVs cannot achieve high-throughput performance. In addition, the mobility of the buoy nodes in the UAV flight period is not considered in this paper. In reality, the position of the buoy node is highly susceptible to water velocity, wind speed, and other environmental and human influences, which means that the update and prediction mechanism for the buoy’s position and wireless traffic can further improve the stability of the system [43]. Therefore, designing a buoy position prediction algorithm, constructing a multi-UAV cooperative network, and coordinating the relationship between hovering and cruising in the UAV cluster provide many opportunities for further research.

In addition, as this paper uses the SCA technique to approximate nonconvex subproblems as convex problems, the approximate accuracy of the solution in each block cannot be guaranteed, and it will directly affect the convergence speed and the quality of the obtained suboptimal solution. Analyzing the geometric characteristics and quadratic structure of the objective function to find a concave surrogate function that has better curvature than the first-order Taylor expansion is a promising direction to explore the global optimal solution in the joint UAV trajectory and power control problem.

## Figures and Tables

**Table 1:** List of main symbols.

K	Set of buoys
$K_{BD}$	Set of buoys in direct transmission mode
$K_{BR}$	Set of buoys in relay transmission mode
*T*	UAV flying period
Δ	Length of sampling slot
*N*	Number of sampling slots
$G_{k}$	Horizontal coordinate of buoys
*O*	Horizontal coordinate of ST
$U\left[n\right]$	Horizontal coordinate of the UAV at the *n*-th time slot
$V_{\max}$	Maximum speed of the UAV
*H*	Flying altitude of the UAV
$G_{0}$	Channel power gain at the reference distance
$R_{0}$	Reference distance
$\sigma^{2}$	Power noise at the ST receiver
η	RF-to-direct current (DC) energy conversion efficiency
$g^{UB}\left[n\right]$	Channel power gain between the UAV and the ST
$g_{k}^{BU}\left[n\right]$	Channel power gain between the UAV and *k*-th buoy
$g_{k}^{GB}$	Channel power gain between the ST and *k*-th buoy
$Q_{E}$	Available energy for UAV energy broadcasting
$B_{k}\left[n\right]$	Stored energy of the *k*-th BR before the *n*-th time slot
$E_{k}\left[n\right]$	Harvested energy of the *k*-th buoy in the n-th slot
*B*	Bandwidth
$R_{k}^{BU}\left[n\right]$	Data rate from the *k*-th BR to the UAV in the *n*-th slot
$R^{UB}\left[n\right]$	Data rate from the UAV to the ST in the *n*-th slot
$R_{k}^{GB}\left[n\right]$	Data rate between the BRs and the UAV
$R_{thr}$	Data rate threshold of buoys
$R_{k}^{\max}$	Maximum rate between the *k*-th buoy and ST in DT mode
$\delta_{k}\left[n\right]$	*k*-th allocated subslot in the *n*-th slot under the TMDA protocol
$P_{E}\left[n\right]$	Energy transmission power of the UAV during slot *n*
$P_{E}^{\max}$	Peak broadcast power of the UAV
$P_{I}\left[n\right]$	UAV information transmission power during the *n*-th slot
$P_{I}^{\max}$	UAV maximum information forwarding power
$P_{k}\left[n\right]$	*k*-th BR transmitting power in subslot $\delta_{k}\left[n\right]$
$P_{k}^{\max}$	Peak transmission power of BRs
$P_{k}^{ave}$	Average transmission power of the *k*-th BD
